# Heparin-Induced Thrombocytopenia Associated with Massive Intracardiac Thrombosis: A Case Report

**DOI:** 10.1155/2012/257023

**Published:** 2012-03-29

**Authors:** Atheer Ahmed, Hamid AL-Mondhiry, Truman J. Milling, David Campbell

**Affiliations:** ^1^The Department of Emergency Medicine, Killeen Metroplex Hospital, Killeen, TX 76549, USA; ^2^The Division of Hematology-Oncology, The Penn State Milton S. Hershey Medical Center, Hershey, PA 17033-0850, USA; ^3^The Department of Emergency Medicine, The University Medical Center at Brackenridge, Austin, TX 78701, USA; ^4^Department of Surgery, The Penn State Milton S. Hershey Medical Center, Hershey, PA 17033-0850, USA

## Abstract

A 60-years old patient was admitted to a community hospital with septic arthritis. He was treated with antibiotics and subcutaneous unfractionated heparin (UH) was used for venous thromboprophylaxis. After three days, he developed leg deep venous thrombosis and was treated with IV heparin. One day later, the patient developed pulmonary emboli, which was found using ventilation/perfusion scan. He was transferred to the University Hospital for further management. Upon arrival, antibiotic and intravenous UH were continued. Trans-Esophageal Echocardiogram showed a thrombus in the right atrium, a small portion of which extended to the left atrium through a patent foramen ovale. Another large thrombus was noted in the right ventricle, which extended to the pulmonary artery. Review of the patient's medical records revealed a halving of his platelet count three days following the heparin administration. Therefore, HIT seemed very likely. Intravenous UH was stopped and an emergency thrombectomy was performed. ELISA testing of HIT antibodies came *negative*. This made HIT diagnosis unlikely and the patient received dalteparin. A week later, as the platelet count declined again, HIT antibodies' testing using ELISA and C-14 serotonin release was repeated, and both assays were *positive*. Argatroban was restarted and the platelet count normalized.

## 1. Introduction

Heparin-induced thrombocytopenia (HIT) is an intensely thrombotic, life-threatening disorder caused by platelet-activating antibodies directed against a complex of platelet factor 4 (PF4) and heparin [1, 2]. HIT, which may be one of the most common drug-induced disorders with a frequency 1–5% [3], is at times a challenging diagnostic and therapeutic problem. HIT is a clinical diagnosis supported by laboratory evidence of a significant serum titer of PF4-heparin complex antibodies [4]. However, physicians often face difficulties establishing a firm diagnosis, and if treatment is delayed, patients may suffer devastating complications.

 In this case report, we describe a patient with HIT and catastrophic intracardiac thrombosis who underwent emergency thrombectomy using cardiopulmonary bypass (CPB) during which a large dose of unfractionated heparin (UFH) must be used. Management was further complicated by a false negative ELISA test for PF4-heparin antibodies. Subsequently, delayed and improper management led to persistence of thrombocytopenia, which prompted retesting using both an immunological test (ELISA) and a serotonin release assay (SRA). At this time the diagnosis was firmly established with positive results from both tests.

## 2. Case Report

A 60-year-old man with no prior history of recent exposure to heparin was admitted to a community hospital with septic arthritis of his right knee. He was treated with antibiotics, and subcutaneous UFH was used for venous thromboprophylaxis. Three days after admission, he developed lower extremity deep venous thrombosis (DVT) and was treated with intravenous (IV) heparin and an inferior vena cava (IVC) filter placement. An initial ELISA test for heparin-PF4 antibodies was ordered. One day later, the patient developed sudden onset shortness of breath with pre-syncope. A ventilation/perfusion lung (V/Q) scan indicated a high probability for bilateral pulmonary emboli (PE). He was transferred to the University hospital for further management.

**Figure 1:**
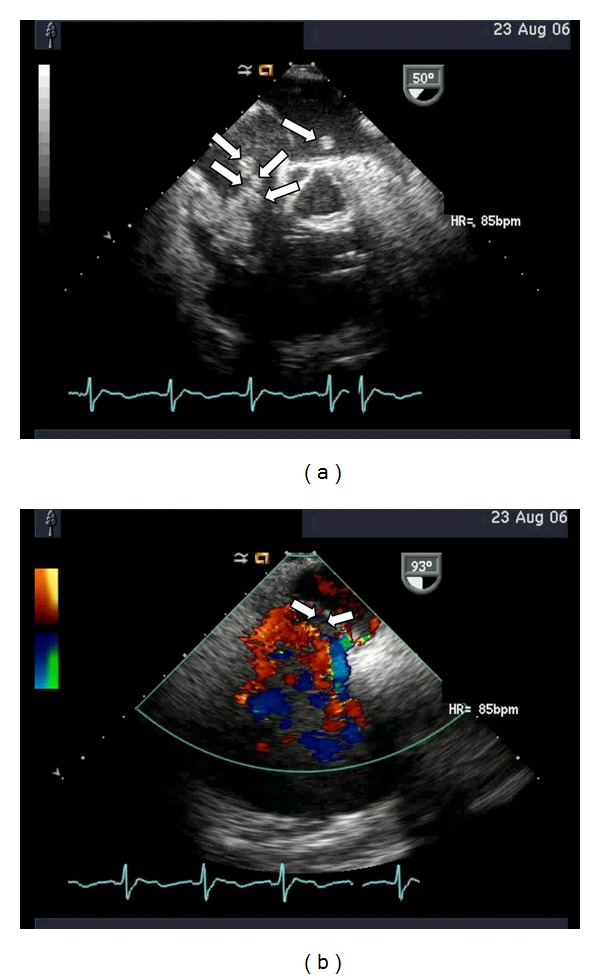
(a) TEE image showing four-chamber view with widely dilated right atrium and right ventricle that are filled with thrombi (indicated by arrows), (b) TEE image showing the patent foramen ovale that was created because of increased intra-atrial pressure in the right atrium (indicated by arrows).

Upon arrival, he appeared clinically stable, and antibiotic and intravenous UFH were continued. Transesophageal echocardiogram (TEE) showed a thrombus in the right atrium (RA), a small portion of which extended to the left atrium through a patent foramen ovale. Another large thrombus was noted in the right ventricle (RV), which extended to the pulmonary artery (Figure 1). 

**Figure 2:**
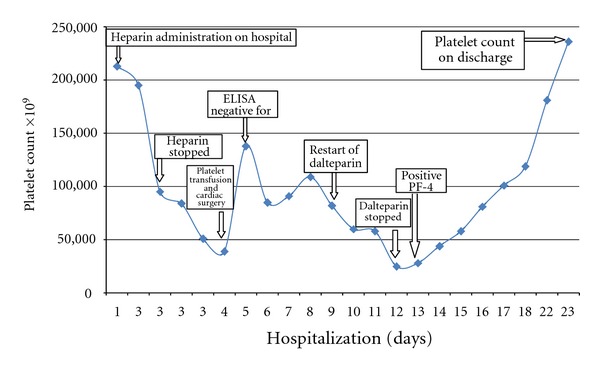
Platelet count trend in correlation to various events in the clinical history.

Review of the patient's medical records, from the referring hospital, revealed a significant decrease of his platelet count (from 213,000 × 10^9^/L to 95,000 × 10^9^/L) three days following the heparin administration (Figure 2). At this point HIT seemed very likely. Intravenous UFH was stopped, and an emergency thrombectomy was performed. Because of the high likelihood of HIT, a special procedure was implemented to prepare the patient for CPB during which a large dose of UFH is used. Immediately before connecting the patient to the CPB, he received tirofiban, a nonpeptide GPIIb-IIIa inhibitor with an intravenous initial rate of 0.4 microgram per kilogram per minute for 30 minutes and then continued at 0.1 microgram per kilogram per minute. The surgery was completed using UFH. During surgery, large thrombi were noted in the RA and around the junction of the inferior vena cava (IVC). A large thrombus was also noted in the main pulmonary artery, which extended to the right and left pulmonary arteries. The IVC filter, which was placed earlier in the course, was found in the RV just below the pulmonary artery.

Postoperatively, the patient was started on an IV infusion of argatroban, a direct thrombin inhibitor, 2 microgram per kilogram per minute, and the dose was adjusted to keep his activated partial thromboplastin time (aPTT) at one and half to two and half times control. The initial ELISA test for PF4-heparin antibodies was reported negative after three days of his admission, with a titer of less than 0.4 units. Based on this result, the treating physician thought the clinical diagnosis of HIT was unlikely because the ELISA test is highly sensitive with a very high negative predictive value. Consequently, argatroban was stopped, and the patient received dalteparin, a low molecular weight heparin (LMWH), 100 units per kilogram subcutaneously every 12 hours.

Two days later, his platelet count decreased (Figure 2), and another ELISA test and C^14^-serotinin release (SRA) assay for PF4-heparin antibodies were performed. Both were strongly positive. SRA was 98% (cutoff point is over 20%) and the ELISA titer value was 3.173 units (positive is over 0.4 units). Dalteparin was stopped, and IV infusion of argatroban was restarted. The platelet count rose to about 150,000 × 10^9^/L in four days (Figure 2). Subsequently the patient was discharged on fondaparinux, a Factor Xa inhibitor, 10 mg subcutaneously daily.

Two weeks after discharge, he was readmitted with proximal right upper extremity venous thrombosis at the site of his central venous access catheter. The catheter was placed so that the patient would receive IV antibiotics at home after discharge. It was removed with resolution of his symptoms. His platelet count was in the normal range at that time. The thrombotic event in his right forearm was most likely caused by the central venous access catheter. There was no evidence of an active thrombosis at other sites.

## 3. Discussion

 This case report demonstrates several interesting aspects of HIT. The first problem was the unusual severity and magnitude of the thrombotic complications. The patient developed thrombosis in the right side of the heart and the main pulmonary artery and the thrombus extended to both the right and left pulmonary artery. Because of the significantly increased pressure in the RA, part of the clot protruded through an otherwise closed interatrial septum orifice to the left atrium. Cardiac thrombosis is uncommon in HIT, and such massive thrombosis is distinctly rare only few cases have been reported [5, 6].

 The second problem was the diagnostic confusion caused by a false negative ELISA test at a time when HIT was a high clinical probability. The ELISA test demonstrates the presence of PF4-heparin antibodies immunologically. It is highly sensitive (>90%) with a high negative predictive value but low specificity (50–93%) [4]. The clinicians taking care of the patient were misled by the negative ELISA test and allowed the use of LMWH, which is contraindicated in patients with HIT.

 Possible explanations for ELISA false negative were either technical problem with the test or that the titer of the antibodies, at that time, was not high enough to exceed the cutoff point of 0.4 units. The C^14^ SRA is highly specific (80–97%) and sensitive (90–98%). Several experts emphasize that there is no “gold standard” test to diagnose HIT, rather, it is a clinical diagnosis supported by laboratory testing [7]. The circumstances of the patient and the clinical probability of HIT are very important in establishing the diagnosis. In retrospect, the clinicians managing the patient should have continued argatroban until the results of repeated dual immunologic (ELISA) and functional testing (C^14^ SRA) became available given his high pretest probability.

 The third difficult problem we faced was how to proceed with CPB surgery in a patient with active HIT and a high thrombotic load. Reexposure to UFH may lead to catastrophic complications. We were unable to use lepirudin, a direct thrombin inhibitor, instead of heparin in the CPB circuit because the special device required to measure Ecarin clotting time (ECT) to monitor the effectiveness of anticoagulation during the surgical procedure [8] was not available. The patient required an urgent life-saving cardiac and pulmonary embolectomy, and there was no option for a time-consuming alternative procedure.

 We decided to use tirofiban, a potent GPIIb-IIIa inhibitor, to maximally inhibit the patient's platelets prior to reexposure to heparin. The rationale for this approach is that if GPIIb-IIIa receptors on platelet membrane are effectively inhibited, reexposure to heparin will not activate platelets. This approach, though considered off-label, has been used in several patients with successful outcome [9–11].

However, more information must be gathered using this approach on a sizeable number of patients before its safety and efficacy can be reasonably determined.
